# Supplementary material for: Hederacolchiside A1 Suppresses Autophagy by Inhibiting Cathepsin C and Reduces the Growth of Colon Cancer
Source: Cancers (Basel). 2023 Feb 16;15(4):1272. doi: 10.3390/cancers15041272 (PMC9953978; doi:10.3390/cancers15041272)

**Figure1B**

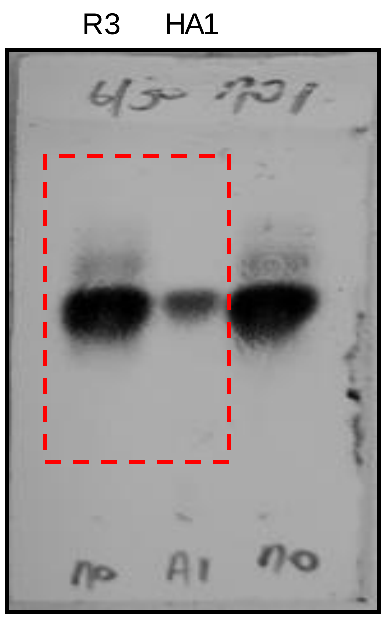


**Figure2A**

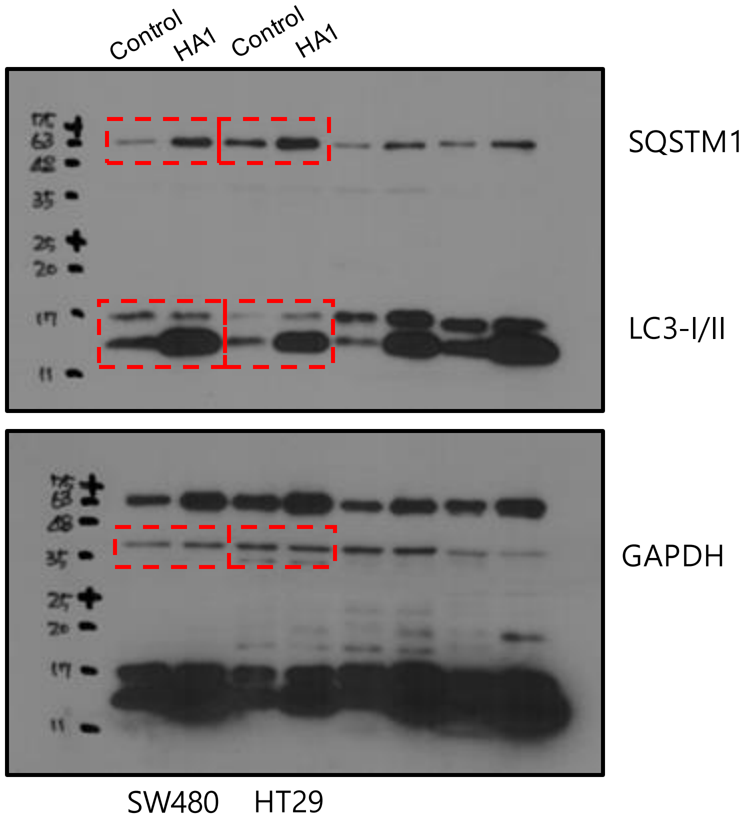

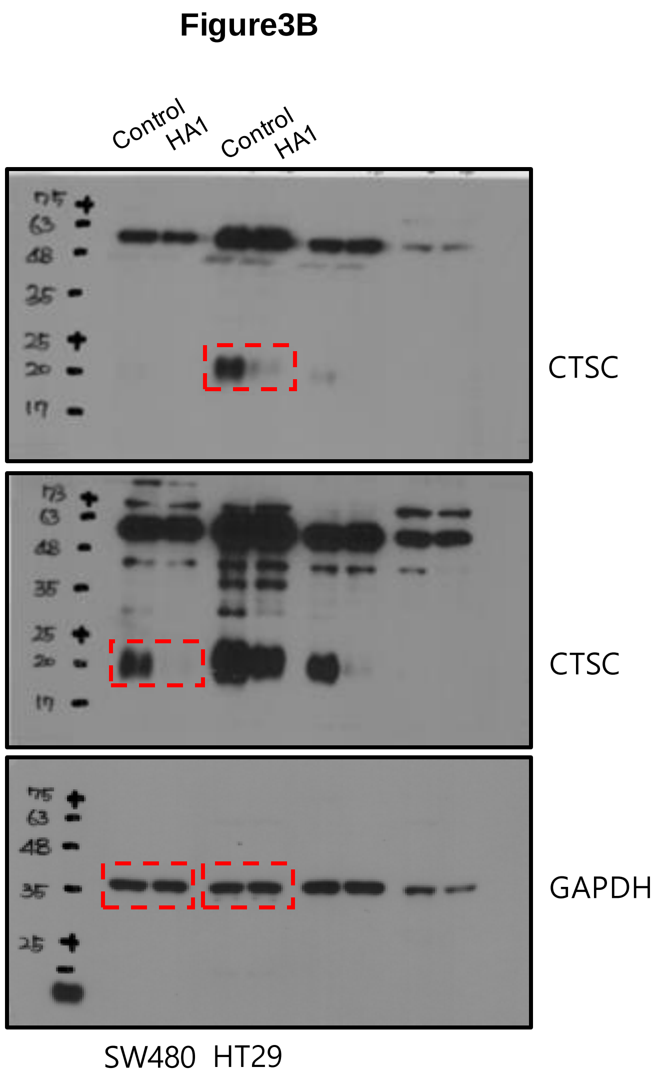
Figure3B
Control
HA1
Control
HA1
CTSC
CTSC
GAPDH
SW480 HT29

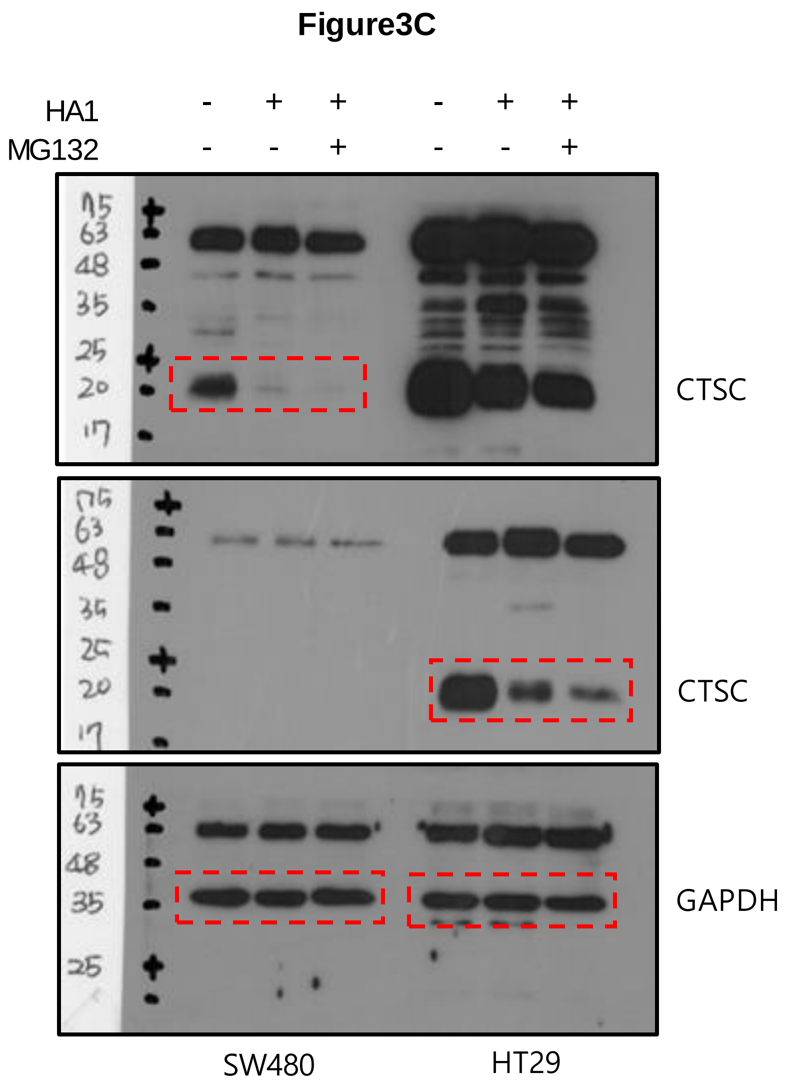
Figure3C
HA1 - + + - + +
MG132 - - + - - +
CTSC
CTSC
GAPDH
SW480
HT29

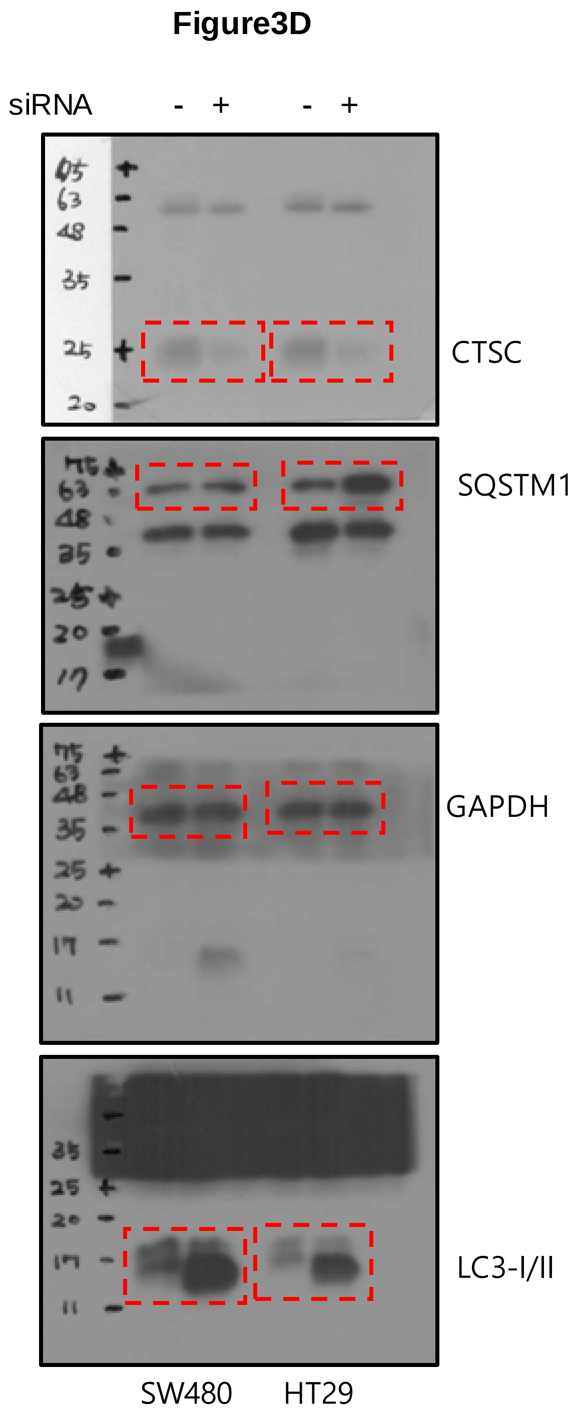
Figure3D
siRNA - + - +
CTSC
SQSTM1
GAPDH
LC3-I/II
SW480 HT29

**Supplementary figure2A**

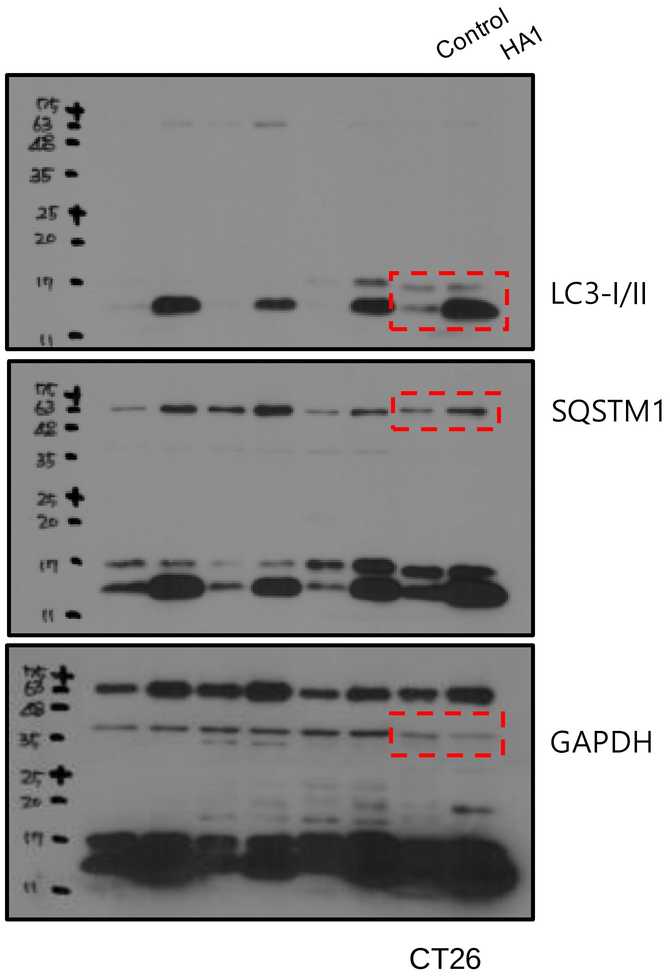


**Supplementary figure2B**

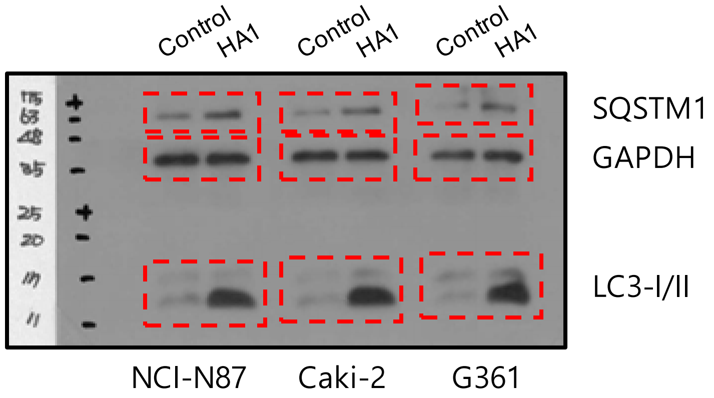


**Supplementary figure2B**

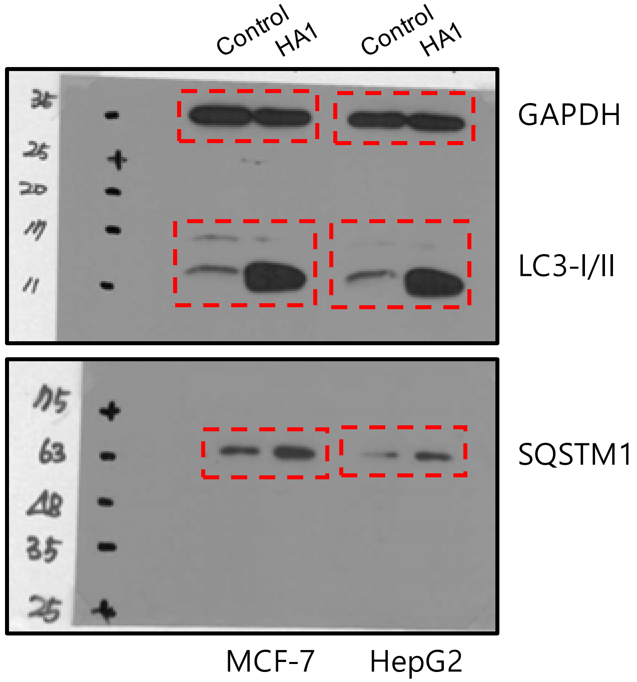

**Supplementary figure3B**

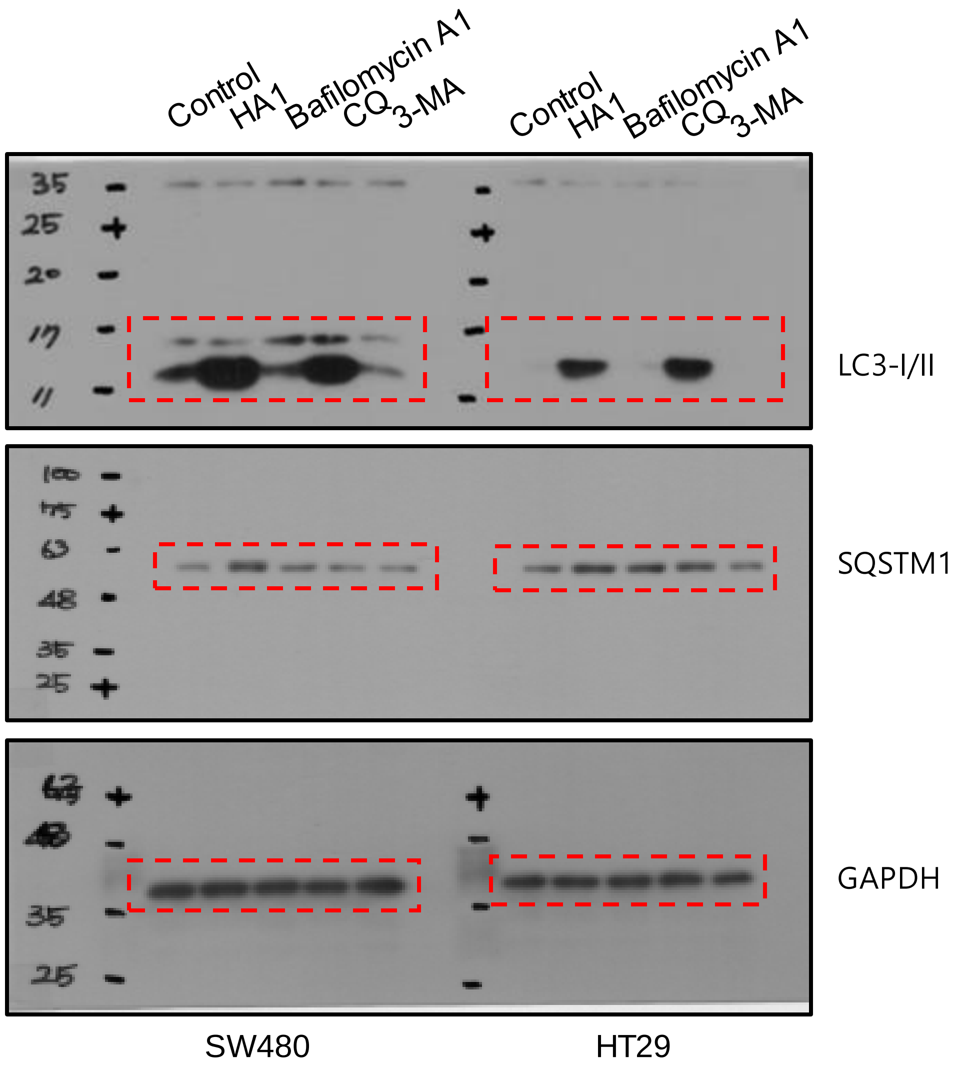

Supplement: Supplementary file 1 [file cancers-15-01272-s001.zip › cancers-2217895-file S1.pdf]
