# Supplementary material for: Hederacolchiside A1 Suppresses Autophagy by Inhibiting Cathepsin C and Reduces the Growth of Colon Cancer
Source: Cancers (Basel). 2023 Feb 16;15(4):1272. doi: 10.3390/cancers15041272 (PMC9953978; doi:10.3390/cancers15041272)
Supplement: Supplementary file 1 [file cancers-15-01272-s001.zip › cancers-2217895-supplementary.pdf]

Supplementary Information

Hederacolchiside A1 suppresses autophagy by inhibiting cathepsin C and reduces the growth of colon cancer

Solbi Kim, Kyung-Ha Lee, Hui-Ji Choi, Eunji Kim, Minju Han, Sora Kang, Heung Jin Jeon, Mi-Young Yun, Gyu-Yong Song, Hyo Jin Lee

Supplementary Figure S1.

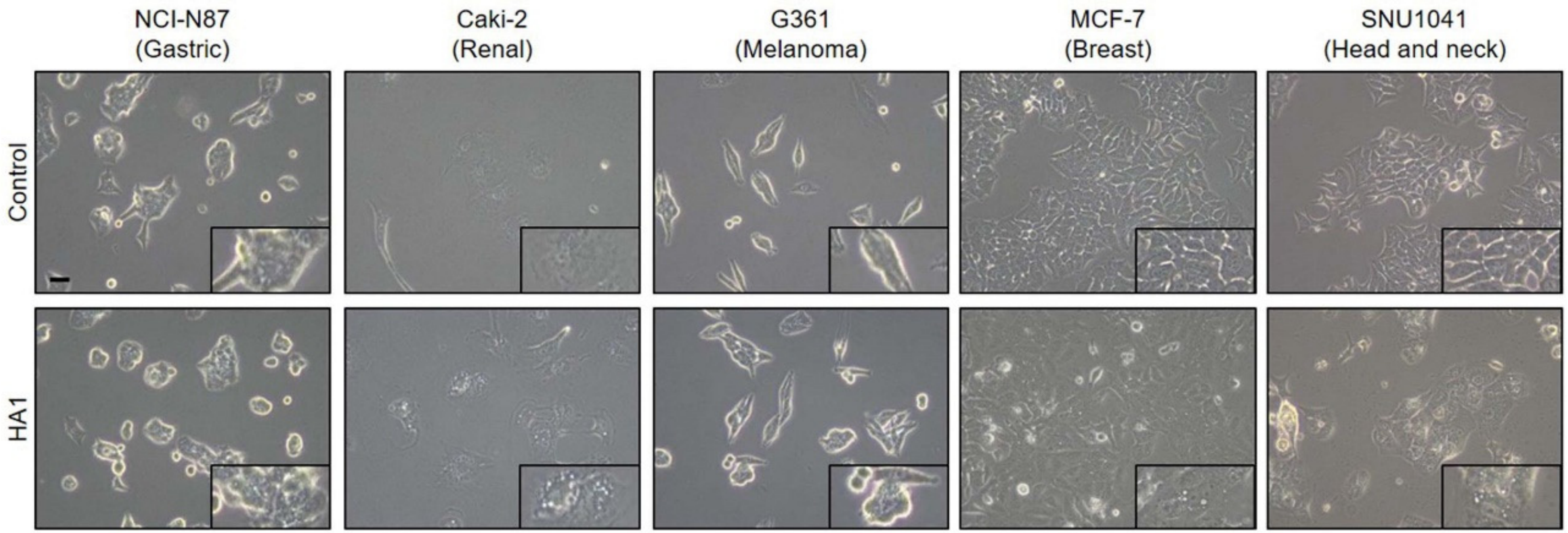

Supplementary Figure S1. HA1 induces vacuolization in other cancer cells. Cancer cells were treated with 10 μM HA1 for 24 h and visualized using an inverted microscope (×200). Scale bar: 20 μm. Inset: higher-magnification view of the upper image (x400).

Supplementary Figure S2.

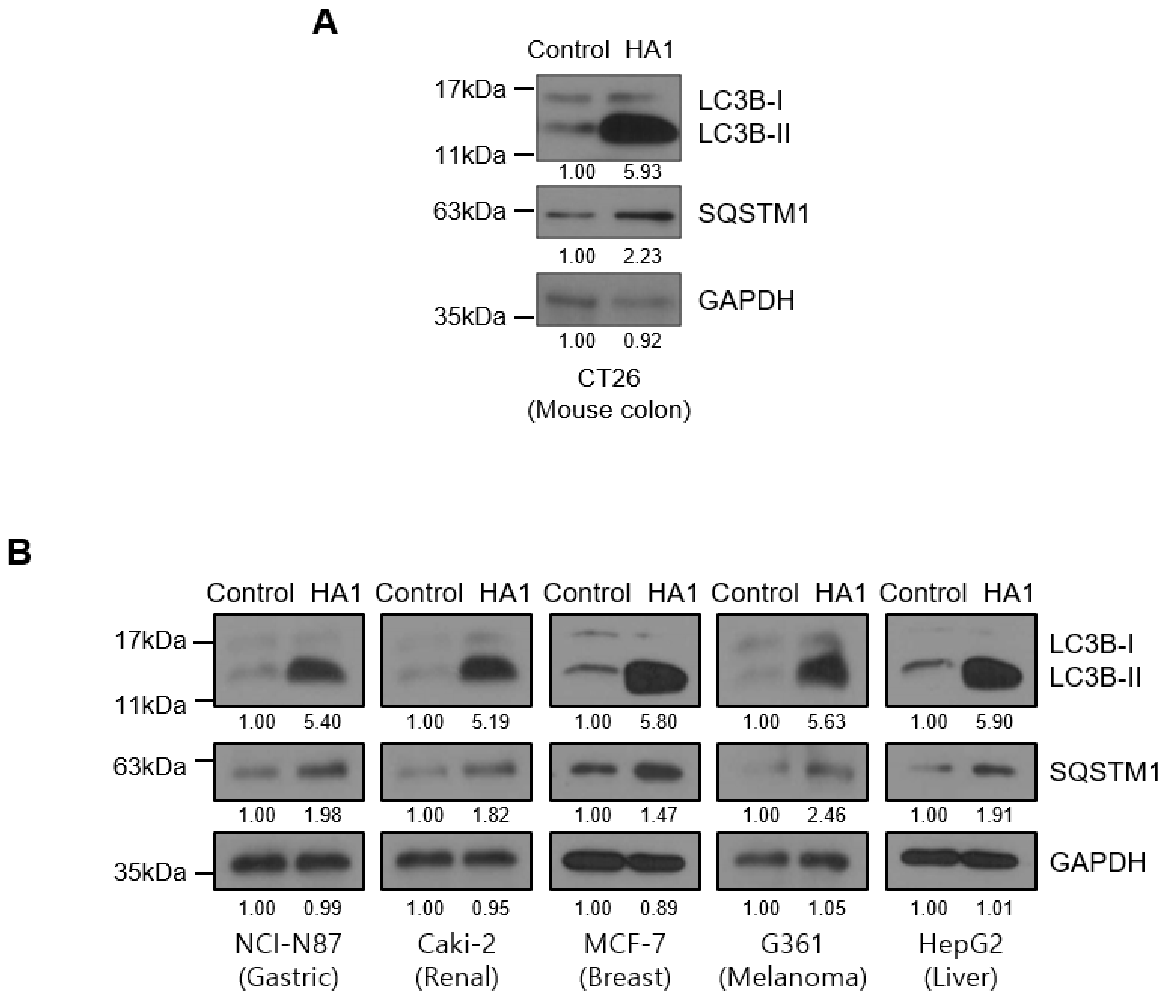

Supplementary Figure S2. HA1 alters autophagy in cancer cell lines. A) B) HA1 increased the LC3B-II and SQSTM1 levels in various cancer cell lines by Western blotting. Western blot bands were measured using Image J software (n = 3).

Supplementary Figure S3.

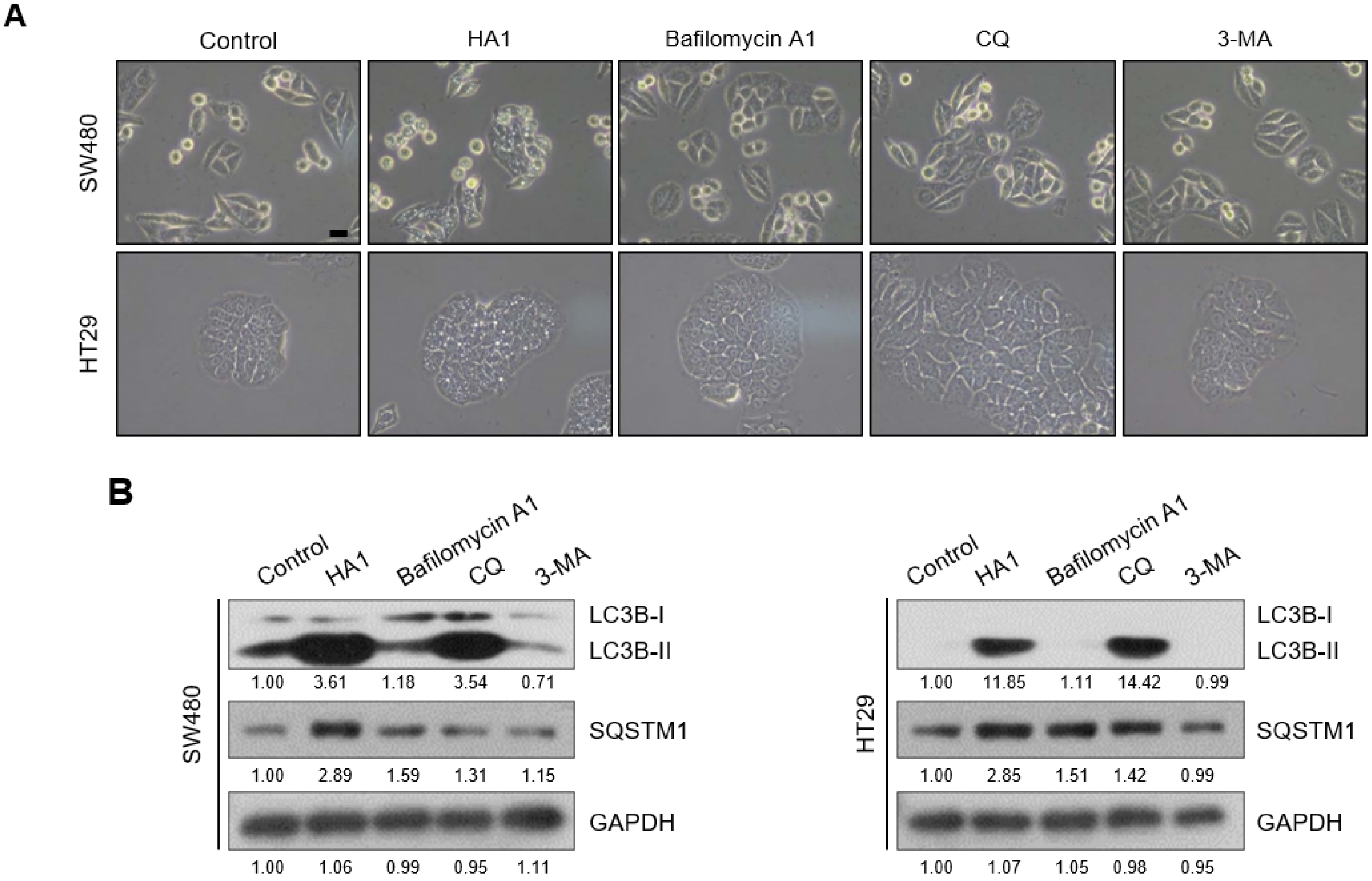

Supplementary Figure S3. HA1 shows distinct characteristics compared with autophagy inhibitors. A) Images (×200) of cells treated with HA1 compared with other autophagy inhibitors (DMSO: 0.1%; HA1: 10 µM; bafilomycin A1: 10 nM; CQ: 40 µM; 3-MA: 2 mM). Scale bar: 20 µm. B) Western blotting of LC3B-I, LC3B-II, SQSTM1, and GAPDH (loading control). Western blot bands were measured using Image J software (n = 3).

Supplementary Figure S4.

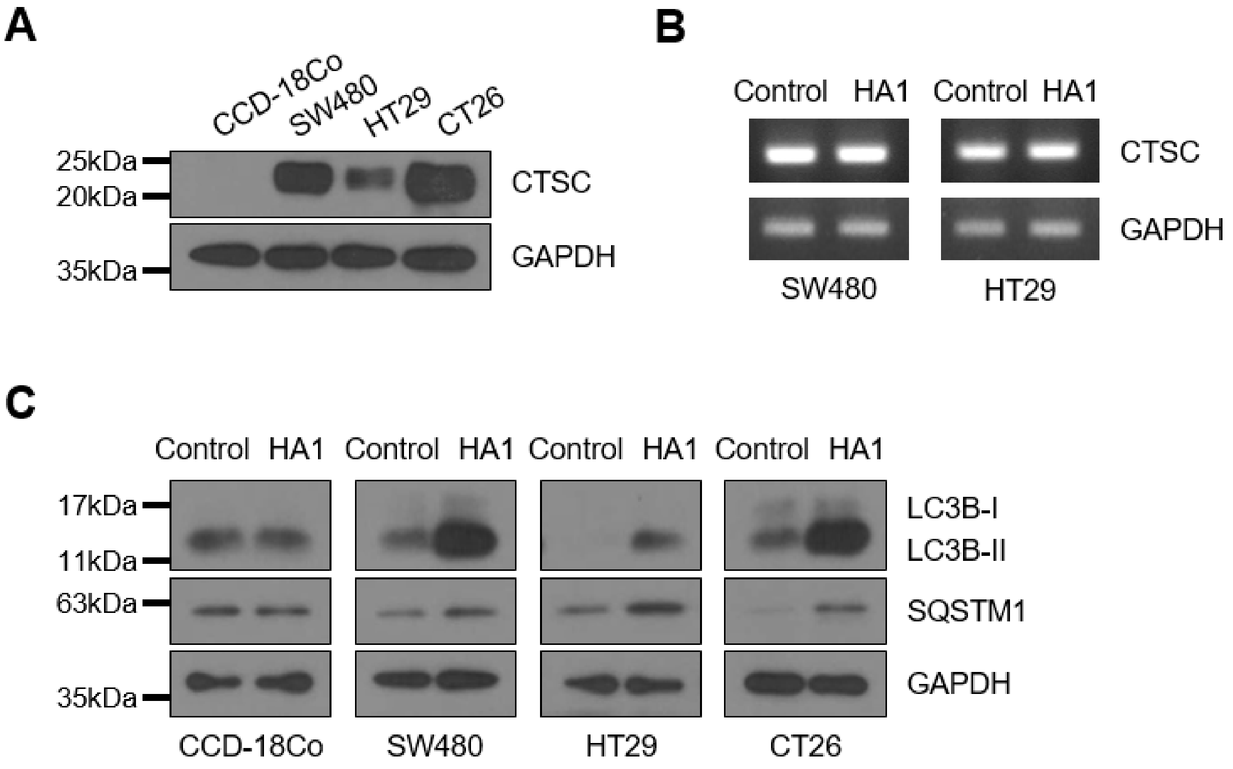

Supplementary Figure S4. Relationship between HA1 and CTSC**.** A) CTSC protein levels were confirmed by western blotting in normal (CCD-18Co isolated from the normal colon tissue) and colon cancer cell lines. B) CTSC mRNA levels after treatment with HA1 for 24 h were confirmed by RT-PCR. C) Autophagy markers evaluated after treatment with HA1 in normal and colon cancer cell lines by western blotting.

Supplementary Figure S5.

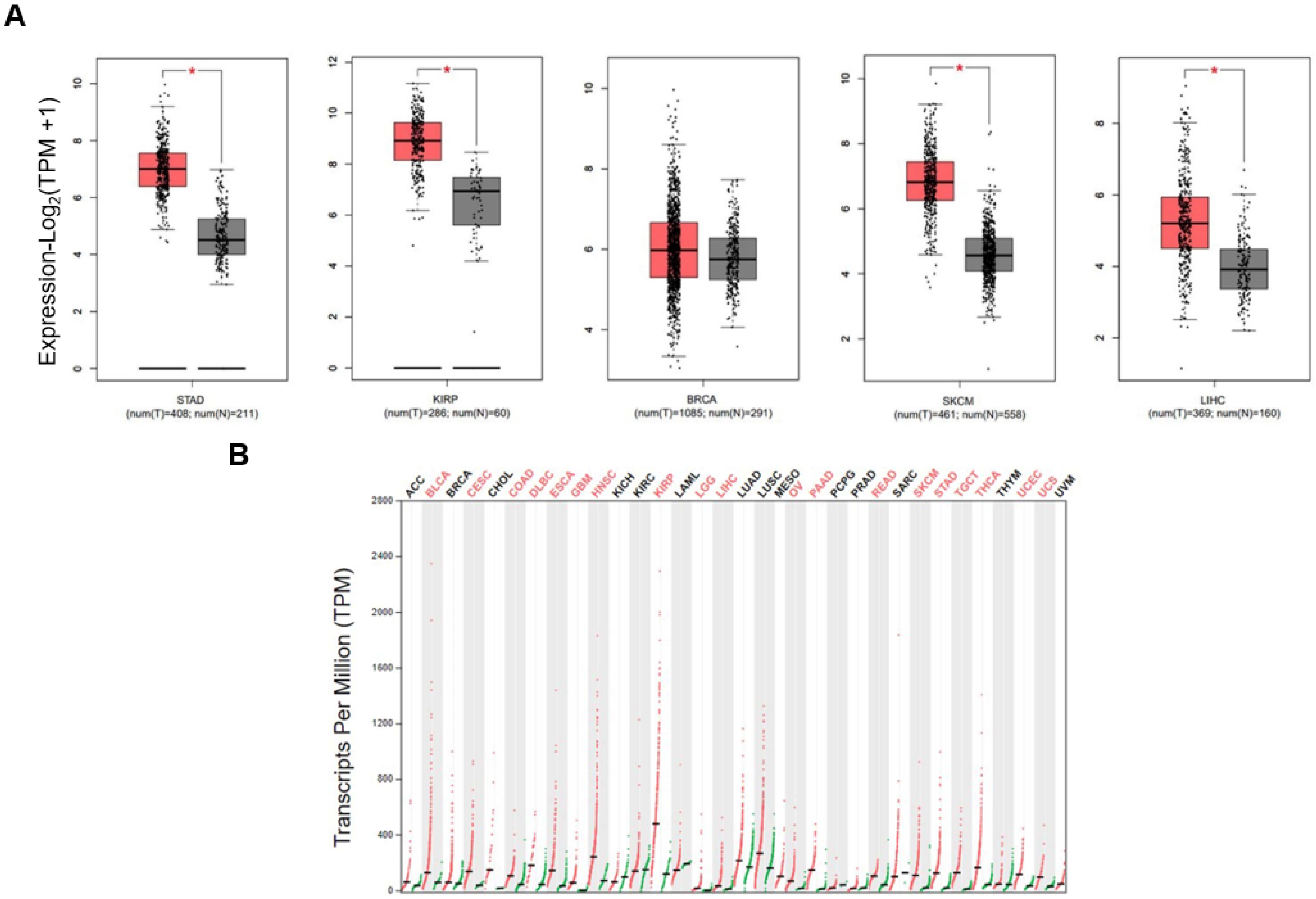

Supplementary Figure S5. CTSC expression in tumor and paired normal tissues (dot plot) in GEPIA. A) CTSC expression in normal (gray box) and cancer (red box) tissues. * $p < 0.01$. STAD, stomach adenocarcinoma; KIRP, kidney renal papillary cell carcinoma; BRCA, breast invasive carcinoma; SKCM, skin cutaneous melanoma; LIHC, liver hepatocellular carcinoma. B) CTSC expression in normal (green dot) and cancer (red dot) tissues. Upregulated CTSC expression in various carcinomas (BLCA, CESC, COAD, DLBC, ESCA, GBM, HNSC, KIRP, LGG, LIHC, OV, PAAD, READ, SKCM, STAD, TGCT, THCA, UCEC, UCS in red color). Normal Genotype-Tissue Expression (GTEx, green) and tumor (red) data were matched. ACC; adrenocortical carcinoma; BLCA, bladder urothelial carcinoma; CESC, cervical squamous cell carcinoma and endocervical adenocarcinoma; CHOL, cholangiocarcinoma; COAD, colon adenocarcinoma; DLBC, lymphoid neoplasm diffuse large B-cell lymphoma; ESCA, esophageal carcinoma; GBM, glioblastoma multiforme; HNSC, head-and-neck squamous cell carcinoma; KICH, kidney chromophobe; KIRC, kidney renal clear cell carcinoma; KIRP, kidney renal papillary cell carcinoma; LAML, acute myeloid leukemia; LGG, brain lower grade glioma; LIHC, liver hepatocellular carcinoma; LUAD, lung adenocarcinoma; LUSC, lung squamous cell carcinoma; MESO, mesothelioma; OV, ovarian serous cystadenocarcinoma; PAAD, pancreatic adenocarcinoma; PCPG, pheochromocytoma and paraganglioma; PRAD, prostate adenocarcinoma; READ, rectum adenocarcinoma; SARC, sarcoma; SKCM, skin cutaneous melanoma; STAD, stomach adenocarcinoma; TGCT, testicular germ cell tumor; THCA, thyroid carcinoma; THYM, thymoma; UCEC, uterine corpus endometrial carcinoma; UCS, uterine carcinoma; UVM; uveal melanoma.

Supplementary Figure S6.

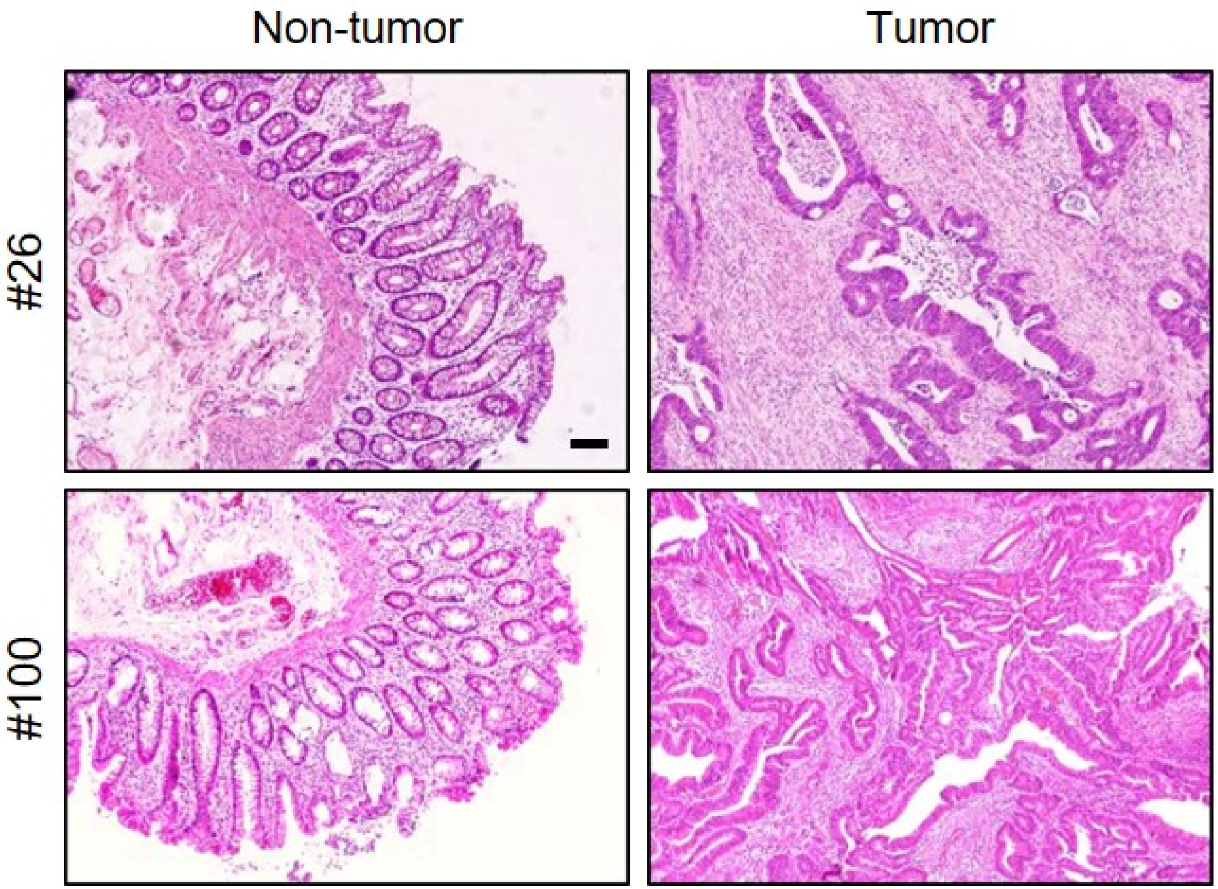

Supplementary Figure S6. Images of the colon cancer patient-derived tissues used to culture organoids. Hematoxylin and eosin staining of adjacent non-tumor and tumor tissues (×100). Non-tumor (#26 and #100) colorectal mucosa showing crypts composed of columnar and goblet cells surrounded by the lamina propria sitting on the muscularis mucosa. Tumor (#26 and #100) colorectal mucosa showing varying size of gland forming tall columnar cells with cytologic atypia surrounded by desmoplastic fibrous stroma. Scale bar: 50 μm.

Supplementary Table S1.

Supplementary Table S1. Characteristics of the colon cancer patients who provided tissues for organoid formation.

Number	Sex	Age	Histopathologic type	Histologic grade	pStage
CC #26	F	70	Adenocarcinoma	G2	pT3N0M0
CC #100	F	44	Adenocarcinoma	G2	pT3N0M0

G, grade; pStage, pathologic stage
